# West Nile Virus–associated Flaccid Paralysis

**DOI:** 10.3201/eid1107.040991

**Published:** 2005-07

**Authors:** James J. Sejvar, Amy V. Bode, Anthony A. Marfin, Grant L. Campbell, David Ewing, Michael Mazowiecki, Pierre V. Pavot, Joseph Schmitt, John Pape, Brad J. Biggerstaff, Lyle R. Petersen

**Affiliations:** * Centers for Disease Control and Prevention, Atlanta, Georgia, USA;; † Centers for Disease Control and Prevention, Fort Collins, Colorado, USA;; ‡ Centennial Neurology, Greeley, Colorado, USA;; § Longmont Clinic, Longmont, Colorado, USA;; ¶ McKee Hospital, Loveland, Colorado, USA;; # Colorado Department of Health and Environment, Denver, Colorado, USA

**Keywords:** poliomyelitis, West Nile virus, respiratory failure

## Abstract

The causes and frequency of acute paralysis and respiratory failure with West Nile virus (WNV) infection are incompletely understood. During the summer and fall of 2003, we conducted a prospective, population-based study among residents of a 3-county area in Colorado, United States, with developing WNV-associated paralysis. Thirty-two patients with developing paralysis and acute WNV infection were identified. Causes included a poliomyelitislike syndrome in 27 (84%) patients and a Guillain-Barré–like syndrome in 4 (13%); 1 had brachial plexus involvement alone. The incidence of poliomyelitislike syndrome was 3.7/100,000. Twelve patients (38%), including 1 with Guillain-Barré–like syndrome, had acute respiratory failure that required endotracheal intubation. At 4 months, 3 patients with respiratory failure died, 2 remained intubated, 25 showed various degrees of improvement, and 2 were lost to followup. A poliomyelitislike syndrome likely involving spinal anterior horn cells is the most common mechanism of WNV-associated paralysis and is associated with significant short- and long-term illness and death.

Acute paralysis associated with West Nile virus (WNV) infection (1–8) has been attributed to Guillain-Barré syndrome (3), a poliomyelitislike syndrome (2,4–6,8), and a generalized myeloradiculitis (1,7). Several reports have described acute respiratory failure occurring with WNV-associated paralysis (5,7). However, the frequency of acute paralysis in WNV neuroinvasive disease remains unknown, and the clinical features of WNV-associated respiratory weakness have not been characterized.

During 2003, Colorado experienced an epidemic of human WNV disease; 2,947 cases were reported to the US Centers for Disease Control and Prevention (CDC) that included 621 neuroinvasive cases and 63 deaths. Acute paralysis was seen in many patients, and in several, acute respiratory failure developed that required emergent intubation. We conducted a population-based assessment of WNV-infected persons in whom acute paralysis developed to describe the clinical features, mechanisms, and short-term outcomes.

## Methods

**Figure A1:**
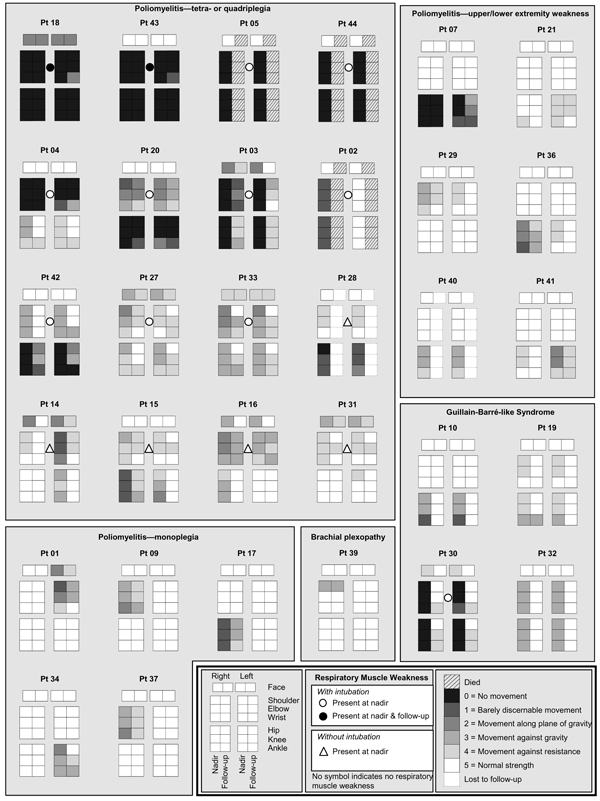
Patterns of weakness at strength nadir and 4 months later in patients with acute paralysis and West Nile virus infection. Shadings indicate strength scoring on manual muscle testing and represent the average strength score of all tested muscles proximally (upper extremity: shoulder adduction/abduction, arm internal/external rotation; lower extremity: hip flexion/extension, thigh adduction/abduction), medially (upper extremity: elbow flexion/extension, pronation/supination; lower extremity: knee flexion/extension), and distally (upper extremity: wrist flexion/extension, finger flexion/extension; lower extremity: ankle plantarflexion/dorsiflexion, foot inversion/eversion, toe flexion/extension) in each limb and the facial muscles. Scores were rounded down to the lowest whole number. Patients with respiratory weakness are indicated by circles (intubated) or triangles (not intubated). Pt, patient.

During the summer and fall of 2003, we identified patients with acute weakness and WNV infection from among the populations of Boulder, Larimer, and Weld counties (combined population ≈724,000) in northern Colorado by active case-finding. We were notified of suspected cases by infection control practitioners and health professionals at 8 hospitals in and around the catchment area and through ongoing state-based surveillance. A suspected case of WNV-associated weakness was defined as muscle weakness in a person of at least 1-point decrement on manual muscle testing by using the Medical Research Council (London, UK) 1–5 scale (Figure A1), respiratory failure requiring intubation that developed <48 hours after presentation, or both. All patients had IgM antibodies detected in acute-phase serum samples by IgM antibody-capture enzyme-linked immunosorbent assay at the Colorado Department of Health Services and Environment (9,10). Remaining acute-phase serum samples from 26 patients were tested by plaque-reduction neutralization assays for antibodies to WNV and St. Louis encephalitis virus at CDC (9,10). All had WNV-specific neutralizing antibody titers ≥1:10, which were at least 4-fold greater than those for St. Louis encephalitis virus.

Patients were approached under the auspices of a public health event, and oral consent was obtained. Results of an initial neurologic examination were recorded, and standardized demographic, clinical, and medical history data were obtained from patient interviews and medical records. Results of serial neurologic examinations were documented on a standardized form.

**Figure A2:**
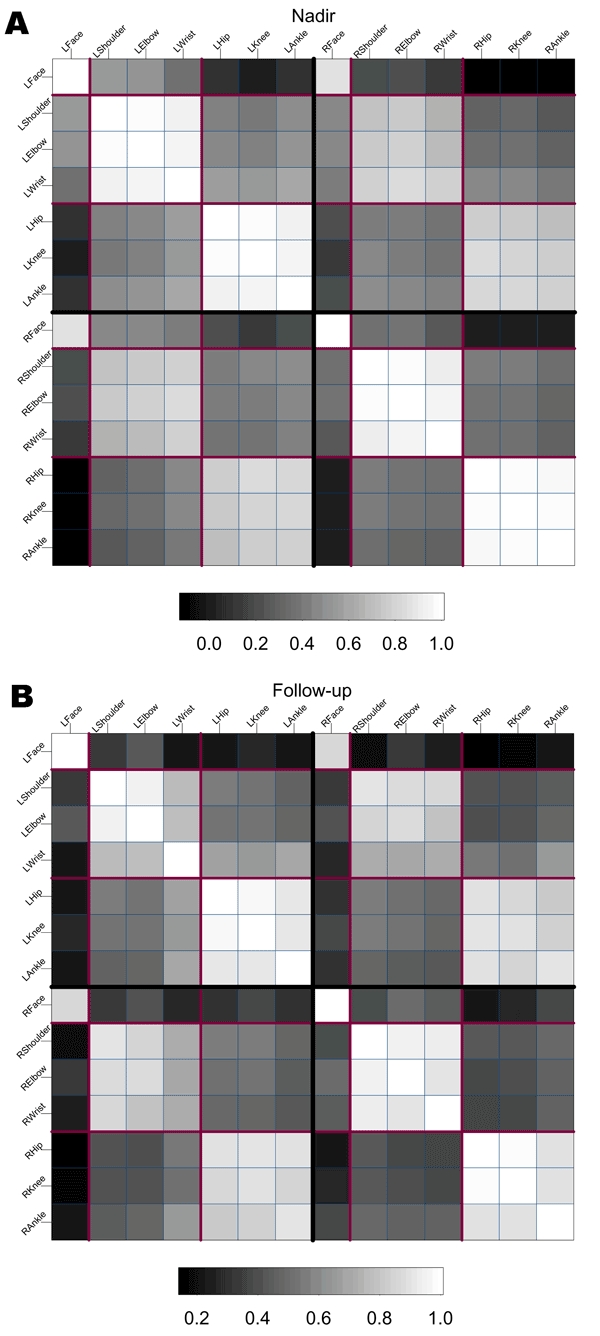
Sample correlation matrices of the 14 strength score measurements at nadir (a) and follow-up (b) for 21 patients with poliomyelitislike syndrome and excluding those who died or were lost to follow-up. Each cell is shaded to represent the correlation between strength scores at the various sites, indicated along the axes, as described and recorded in Figure A1. Darker shading represents lower correlation and white represents perfect (1.0) correlation. The heavy black lines splitting the figure into quadrants separate right and left, and the lighter-shaded lines separate the quadrants into face, arm, and leg measurements. At nadir, consistent correlation is displayed within limbs across the body, with lower correlation between limbs on the same body side; lowest correlation is observed between face and leg. At follow-up, correlations were observed at nadir (e.g., stronger correlation within limbs across body) persist, but associations were weakened somewhat, suggesting greater heterogeneity of strength scores. Weakening of association between distal and proximal limbs suggests distal to proximal improvement. L, left; R, right.

Four months after initial assessment, we repeated the neurologic examinations, and patients or family members completed a self-administered questionnaire that gathered information on functioning in daily activities. Strength scores at 14 locations (Figure A2) were evaluated at nadir and followup by using manual muscle testing scores. A proportional odds model for the strength scores was used to evaluate improvement; anticipated correlation was incorporated by estimating model parameters with generalized estimating equations. Within-patient correlations of the scores were estimated, and all pairwise differences were evaluated for significance by using bootstrap methods (11); adjustment for multiple comparisons was made by using the Bonferroni adjustment. Statistical analyses were performed with SAS version 8.2 (SAS Institute, Cary, NC, USA), S-Plus version 6.2 (Insightful Corp., Seattle, WA, USA), Sudaan version 8.0.2 (Research Triangle Institute, Research Triangle Park, NC, USA), and EpiInfo version 6.04d (CDC, Atlanta, GA, USA).

## Results

**Figure 1:**
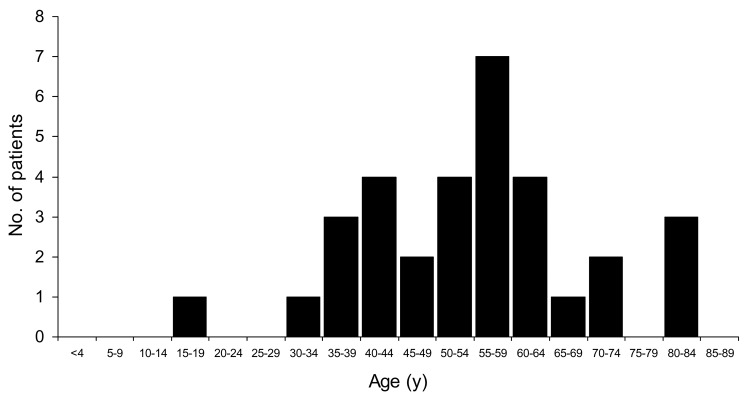
Age distribution of 32 patients with West Nile virus–associated paralysis.

Two hundred nineteen cases of WNV neuroinvasive disease were identified by state-based surveillance in our catchment area; among these, we identified 32 patients with acute paralysis and WNV infection. Eighteen (56%) were male; the median age was 56 years (range 15–84, Figure 1). All but 1 were Caucasian, and 3 (9%) were Hispanic. Sixteen patients (50%) had concomitant encephalitis, 10 (31%) had meningitis, and 6 (19%) had paralysis alone. Those 26 patients with concomitant meningitis or encephalitis represented 12% of the patients identified as having neuroinvasive disease.

Twenty-nine patients were examined by study neurologists within 7 days of weakness onset; 2 patients were evaluated on days 11 and 18 after weakness onset, respectively, and initial neurologic findings for 1 patient were obtained by personal communication (T. Clark, Colorado Pulmonary Associates, Denver, CO, USA). All but 1 of the patients were hospitalized. The median length of stay in the hospital was 17 days (range 2–87 days). Five patients (16%) with encephalitis were immunocompromised: 2 had prior liver transplants, 2 had hematologic malignancies, and 1 was receiving immunosuppressive medication for rheumatoid arthritis. One patient had insulin-dependent diabetes mellitus. Twenty-six (81%) had no prior medical problems.

Twenty-seven patients (84%) had asymmetric weakness consistent with a poliomyelitislike syndrome, 4 (13%) had symmetric ascending weakness with sensory abnormalities consistent with the acute inflammatory demyelinating polyradiculoneuropathy form of Guillain-Barré syndrome, and 1 had scapular winging and shoulder abduction weakness consistent with a long thoracic nerve paralysis.

### Poliomyelitislike Syndrome

The incidence of poliomyelitislike syndrome was 3.7/100,000. Associated signs and symptoms are shown in Table 1. Two patients had weakness in the absence of other systemic features of infectious illness. Among 25 patients with systemic signs or symptoms listed in Table 1, including 4 in which weakness was concurrent with illness onset, the median interval between illness onset and weakness onset was 3 days (range 0–18). All but 1 patient had other neurologic features suggestive of acute WNV infection (Table 1). Patterns of weakness (Figure A1) included acute monoplegia (weakness or paralysis of 1 limb, n = 5); asymmetric upper (n = 1) or lower (n = 5) extremity weakness; and generalized asymmetric tetraplegia or quadriplegia (asymmetric weakness in ≥3 limbs, n = 16). Deep tendon reflexes in affected limbs were diminished or absent. Nineteen patients (70%) with poliomyelitislike syndrome had cranial nerve involvement, which included unilateral (n = 2) or bilateral (n = 8) facial paralysis, extraocular muscle weakness (n = 4), dysphagia (n = 13), dysarthria (n = 6), and vocal cord paralysis (n = 2). Limb weakness by strength testing or subjective patient interpretation progressed to its lowest point (nadir) within 24 hours in 24 patients (88%), with a range of <6 hours to 3 days. Two patients reported sensory deficits (subjective numbness or paresthesias), and 16 (60%) reported pain in affected limbs preceding onset of weakness. Paralysis due to WNV infection in 2 patients occurred exclusively in limbs with prior lower motor neuron dysfunction due to lumbar disc herniation (previously resolved with discectomy).

**Table 1:** Signs and symptoms in 32 patients with West Nile virus (WNV)–associated paralysis

Systemic sign or symptom	Acute infection, N = 32		4-month followup, N = 27	
	n	(%)	n	(%)
Fever (temperature ≥38°C)	29	(91)	0	
Nausea with or without vomiting	26	(81)	0	
Headache	28	(88)	5	(19)
Altered mental status	16	(50)	0	
Meningismus	10*	(31)	0	
Rash	4	(13)	0	
WNV-associated neurologic features				
Tremor	21†	(66)	8	(25)
Myoclonus	15	(47)	2	(6)
Parkinsonism	8	(25)	2	(6)
Cerebellar ataxia	3	(9)	2	(6)
Limb atrophy	0		17	(53)

*Includes 2 patients with Guillain-Barré–like syndrome and 1 patient with long thoracic nerve weakness. †Includes 2 patients with Guillain-Barré–like syndrome.

**Figure 2:**
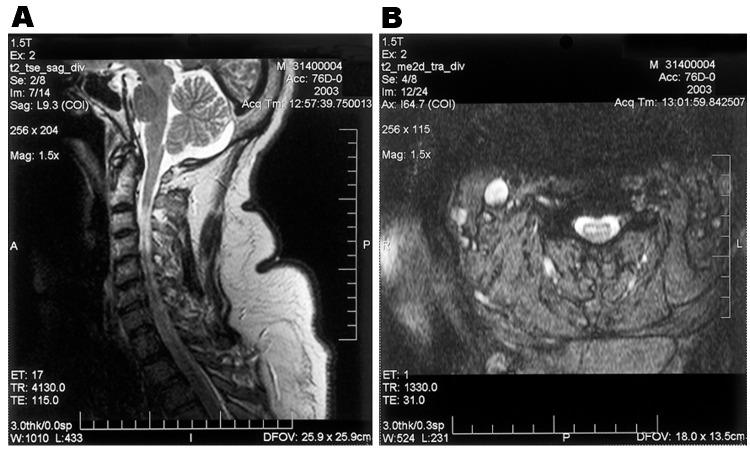
Saggital (A) and axial (B) T2-weighted magnetic resonance images of the cervical spinal cord in a patient with acute asymmetric upper extremity weakness and subjective dyspnea. A shows a diffuse cervical cord signal abnormality, and B shows an abnormal signal in the anterior horn region.

Electromyography/nerve conduction studies performed on 14 patients with poliomyelitislike syndrome suggested a motor axonopathy and/or an anterior horn cell process. Preservation of voluntary motor unit potentials was observed in all tested myotomes in 10 patients, and voluntary motor unit potentials were absent in some myotomes in 4 patients. Fourteen patients with poliomyelitislike syndrome underwent magnetic resonance imaging (MRI) of the brain (n = 6), spine (n = 2), or both (n = 6). Two displayed focal lesions of the basal ganglia, thalami, and brainstem bilaterally on T2- and diffusion-weighted sequences, and 4 (including 3 who did not undergo electromyography/nerve conduction studies) had signal abnormalities in the anterior cord and ventral roots on T2- and diffusion-weighted sequences, suggesting anterior horn cell involvement (Figure 2).

### Guillain-Barré–like Syndrome

Four patients had symmetric, ascending weakness with sensory symptoms suggestive of the acute inflammatory demyelinating polyradiculoneuropathy form of Guillain-Barré syndrome (Figure A1). All 4 had symptoms listed in Table 1 before weakness onset (range 2–12 days), 2 had meningitis before onset of weakness, and 2 had tremors. Initial deep tendon flexes were diminished or absent in all patients. Pain in affected limbs preceded weakness in 2 patients. The median interval between weakness onset and nadir was longer in patients with Guillain-Barré–like syndrome (4 days) than those with poliomyelitislike syndrome (1 day) (p<0.005, Wilcoxon rank sum test). One patient with Guillain-Barré–like syndrome had facial diplegia.

Electromyography/nerve conduction studies were carried out in 3 patients with Guillain-Barré–like syndrome, and all showed findings consistent with a predominantly demyelinating sensorimotor neuropathy. In patients with Guillain-Barré–like syndrome, voluntary motor unit potentials were preserved in tested myotomes.

### Brachial Plexus Neuropathy

One patient with meningitis exhibited apparent, isolated, long thoracic nerve weakness with scapular winging and shoulder abduction weakness but without additional limb involvement. This patient also had pain before onset of weakness.

### Respiratory Involvement

Twelve patients (39%), including 1 patient with Guillain-Barré–like syndrome, developed acute respiratory weakness that required endotracheal intubation and eventual tracheostomy (Table 2). In 10 patients, diaphragmatic paralysis, shown by hemidiaphragmatic elevation on chest radiograph, or pulmonary function test results with a restrictive pattern and retention of carbon dioxide, suggested neuromuscular respiratory failure. These studies were not available for the remaining 2 patients. Five additional patients with poliomyelitislike syndrome had dyspnea and showed evidence of diaphragmatic weakness by chest radiograph and pulmonary function test results, but were not intubated. Three patients died of respiratory failure 7, 27, and 35 days after intubation, after ventilatory support was withdrawn. All patients except the one with Guillain-Barré–like syndrome had encephalitis. Patients in whom dysarthria, dysphagia, or both developed were more likely to experience subsequent respiratory failure (odds ratio [OR] 62, p<0.0001, Fisher exact test). The interval between onset of these bulbar symptoms and intubation was ≤1 day in 9 of 12 patients (range <1–6 days). Facial nerve paralysis without other cranial nerve abnormalities was not associated with subsequent respiratory failure (Table 2).

**Table 2:** Presence of dysarthria/dysphagia, facial weakness, encephalitis, and immunocompromised status among patients with and without respiratory failure due to West Nile virus infection

Variable	Respiratory failure (N = 12*)	No respiratory failure (N = 20)	Odds ratio	p value†
Dysarthria/dysphagia	11	3	62	<0.0001
Immunocompromised status	4	0	Undefined	0.01
Encephalitis	12	5	Undefined	<0.0001
Facial nerve weakness	6	5	3	0.25

*Includes 1 patient with respiratory weakness secondary to Guillain-Barré–like syndrome. †Fisher exact test.

Immunocompromised patients and patients with encephalitis were more likely to experience respiratory failure (Table 2). Pattern of limb weakness was not predictive of respiratory failure; 3 patients had initial upper extremity involvement, 4 had initial lower extremity involvement, and 5 had more diffuse generalized involvement before respiratory failure.

### Cerebrospinal Fluid (CSF) Parameters

Lumbar puncture was performed on 30 patients. They had a median CSF leukocyte count of 108 cells/mm^3^ (range 0–740 cells/mm^3^), a median CSF protein level of 98 mg/dL (range 27–138 mg/dL), and a median CSF glucose level of 55 mg/dL (range 36–135 mg/dL). Pleocytosis was lymphocytic in 14 patients, neutrophilic in 13, and unknown in 3. Among recorded cell differentials (n = 27), neutrophils were predominant in CSF collected on or before the day of weakness onset (10/14, 71%). CSF collected after the time of weakness onset was more often lymphocytic (10/13, 77%, OR 8.3, p = 0.03). Three of 4 patients with Guillain-Barré–like syndrome had lumbar punctures; 2 had cytoalbuminologic dissociation (elevated protein levels without pleocytosis) commonly seen in Guillain-Barré syndrome, and 1 had pleocytosis with an elevated protein level. The median CSF leukocyte count and protein level (64 cells/mm^3^ and 100 mg/dL, respectively) in patients with respiratory failure were not significantly different from those without respiratory failure (117 cells/mm^3^ and 96 mg/dL, each p = 0.5, Wilcoxon rank sum test).

### Short-term Outcomes

Three patients with poliomyelitislike syndrome died in the hospital after withdrawal of ventilatory support. Of the surviving patients, 13 were discharged and 15 (including 1 with Guillain-Barré–like syndrome) were admitted to long-term care facilities. The patient with long thoracic nerve palsy was not hospitalized. At 4 months, 2 patients with poliomyelitislike syndrome were lost to followup. Of 15 patients sent to long-term care facilities, 5 with poliomyelitislike syndrome and 1 with Guillain-Barré–like syndrome remained in these facilities, and the remaining 9 had been discharged (median length of stay 40 days, range 17–106). No persons initially discharged were readmitted. Of 21 patients employed or in school before illness, 9 were working full-time or part-time at the 4-month followup. Seventeen required continuing physical therapy.

Four of the 27 reassessed patients (patients 4, 18, 20, and 43), all with poliomyelitislike syndrome, showed almost no improvement in strength of involved muscles. Two patients with Guillain-Barré–like syndrome regained baseline strength. Although the remainder showed general improvement by the proportional odds model (p<0.001), none had returned to baseline status (Figure A1). In patients with poliomyelitislike syndrome undergoing electromyography studies, the absence of voluntary motor unit potentials was associated with lack of strength improvement in involved myotomes. Neurologic signs at 4 months are shown in Table 1. Three patients (all with respiratory paralysis) were nonambulatory; 12 of 24 ambulatory patients, including 2 with Guillain-Barré–like syndrome, required a wheelchair (n = 4), walker (n = 3), or canes or crutches (n = 5).

Pairwise comparisons of the correlations among patients with poliomyelitislike syndrome showed that at both times, weakness was more likely to be of greater severity in comparable limbs across the body (e.g., both arms, both legs) than in limbs on the same side (e.g., right arm and leg); facial weakness was strongly correlated with ipsilateral arm weakness (Figure A2). Limbs improved distally to a greater extent than proximally.

Of 9 surviving patients who had respiratory failure, 2 remained intubated at 4 months, and 7 displayed various degrees of improvement (Figure A1); however, none had returned to baseline strength or level of function. The median duration of intubation for the 7 extubated patients was 49 days (mean 66 days, range 21–135).

## Discussion

The overall incidence of WNV-associated paralysis in this population was 4.3/100,000, with an incidence of poliomyelitislike syndrome of 3.7/100,000, which is comparable to that of paralysis seen during epidemics of poliovirus disease (12). During 2003, 219 cases of neuroinvasive disease were reported in this 3-county area of Colorado (13), of whom we estimate that flaccid paralysis developed in 12%. A total of 2,773 cases of WNV neuroinvasive disease were reported in the United States during the 2003 epidemic (13); ≈330 cases of WNV-associated neuromuscular weakness may have occurred in the United States during this period.

In contrast to persons typically considered at risk for WNV encephalitis alone, our patients were relatively young, with a third-quartile age of 61 years (Figure 1), and healthy, with >80% having no prior medical conditions. Many patients required prolonged hospitalization and time in rehabilitation facilities and had severe disabilities, which suggests that paralysis caused by WNV infection is associated with considerable lost productivity and incurred healthcare costs. Thus, the long-term economic impact of WNV paralysis needs assessment.

Twenty-seven patients with WNV-associated paralysis had electrophysiologic or neuroimaging evidence of a poliomyelitislike syndrome or a clinical syndrome compatible with this diagnosis. These patients had asymmetric, acute weakness, pleocytosis, and in 14 patients with studies performed, results of electromyography/nerve conduction studies were consistent with motor axonopathy, anterior horn cell involvement, or both. However, 4 patients had clinical, electrophysiologic, and laboratory findings more suggestive of a Guillain-Barré–like syndrome. These patients had characteristic bilateral, symmetric, ascending weakness with associated sensory symptoms, and 2 of 3 patients with available CSF data showed characteristic cytoalbuminologic dissociation. The 3 patients who underwent electrodiagnostic studies had findings consistent with a demyelinating sensorimotor neuropathy typical of Guillain-Barré–like syndrome (3,14). Further electrophysiologic and pathologic characterization of this syndrome is needed because its management may differ from that of poliomyelitis.

Although WNV-associated weakness may occur without other findings suggesting acute WNV disease (14), all but 6 of our patients had meningitis or encephalitis and displayed other WNV-associated neurologic signs, including tremors, myoclonus, and parkinsonism (15–18). A neutrophilic, rather than the more typical lymphocytic, pleocytosis may be seen soon after onset of WNV disease (17) or other viral infections of the central nervous system (19–21). Our patients who had CSF obtained on the day of or shortly after weakness onset were more likely to have a neutrophilic pleocytosis than those with CSF obtained later in their illness. Clinicians should recognize the potential for a neutrophilic predominance in CSF obtained early in the course of WNV neuroinvasive disease.

A generalized asymmetric tetraplegia or quadriplegia was the most common weakness pattern, followed by monoplegia. Consistent with previous reports (22), all patients except 2 with Guillain-Barré–like syndrome demonstrated continued weakness at 4 months, although nearly all had some improvement in strength as indicated by manual muscle testing scores. All 10 patients with a poliomyelitislike illness who had even minimal preservation of motor unit potentials on initial electromyogram improved in strength in associated myotomes at 4 months; 4 patients with no motor unit potentials on initial electromyogram did not improve in strength in these myotomes. Complete destruction of large spinal motor neurons correlating with a completely paralyzed muscle has been observed in poliovirus-associated paralysis (23). Absence of motor unit potentials on electromyography may reflect this loss and may have prognostic value for future strength recovery. However, independent predictors of strength outcome remain unknown.

The facial weakness observed in 40% of patients with poliomyelitislike syndrome and in 1 patient with Guillain-Barré–like syndrome nearly or completely resolved, which is consistent with observations of patients with poliovirus disease and suggests a favorable outcome of this manifestation (24,25). Weakness was more severe in congruent limbs across the body than in ipsilateral limbs, and facial weakness was associated more strongly with arm than with leg weakness, which is consistent with the patchy focal cell destruction demonstrated pathologically in WNV poliomyelitis (26). Improvement in limb strength tended to occur distally to proximally.

Thirty-eight percent of the patients, including all 3 who died, had respiratory failure requiring intubation, and 16% of the patients had dyspnea and diagnostic evidence of neuromuscular respiratory failure but were not intubated. Respiratory failure has been described with WNV-associated paralysis (6–8) but has not been characterized in detail. In 1 patient with Guillain-Barré–like syndrome, respiratory failure developed, a common complication of this syndrome (27). However, the association with limb weakness of a motor neuron or anterior horn cell type, presence of an elevated hemidiaphragm on chest radiograph, and a restrictive pattern of respiratory failure by pulmonary function testing suggest a central, poliomyelitislike etiology for respiratory failure in all other patients. This etiology is supported by the electrodiagnostic findings of lower motor neuron involvement in affected limbs in 5 patients with respiratory failure. Additionally, histopathologic findings in patients with respiratory weakness and WNV infection have demonstrated neuronophagia and leukocytic inflammation of the dorsal motor nuclei of the vagus and glossopharyngeal nerves, which is similar to that seen in the spinal anterior horns (26). Disease due to poliovirus infection has been associated with diaphragmatic, intercostal muscle, and bulbar weakness with respiratory failure (28–31), and poliomyelitislike respiratory insufficiency has been described in infections with other flaviviruses (32).

Lower bulbar dysfunction, specifically dysarthria and dysphagia, was more frequent in patients with respiratory failure; in most, lower bulbar dysfunction followed or was concurrent with limb weakness and preceded respiratory failure by less than a day. Patients with lower bulbar signs and acute limb paralysis require monitoring for respiratory failure. Facial paralysis was not associated with increased risk of respiratory failure, which possibly reflects the neuroanatomic separation of the involved cranial nerve nuclei. Although results of MRI are frequently reported as normal in patients with WNV neuroinvasive disease, detailed images of brainstem and cervical spine are frequently not obtained. Although 4 patients displayed spinal cord and ventral root lesions at involved levels, and 1 patient with respiratory failure had lower brainstem signal abnormalities, detailed brainstem and spinal cord images in our patients were generally not obtained. In patients with acute weakness and bulbar signs, MRI should include the lower brainstem and spinal cord. Although 2 patients remained intubated at 4 months, all other surviving patients with respiratory involvement were extubated, although duration of ventilatory support was often prolonged.

In summary, our findings suggest that involvement of WNV with anterior horn cells, which resulted in a poliomyelitislike syndrome, represents the most common underlying cause of paralysis with WNV infection. In the population assessed by our study, the incidence of paralysis was comparable to that seen during large epidemics of poliovirus infection. Respiratory involvement was a frequent and severe manifestation of this syndrome, with a high degree of illness and death. Thus, early and prominent dysarthria and dysphagia may be predictors of subsequent respiratory failure in this group.
